# Body roundness index and rheumatoid arthritis risk: Evidence from NHANES 1999 to 2020

**DOI:** 10.1097/MD.0000000000046618

**Published:** 2025-12-26

**Authors:** Xin Huang, Wenzhuo Huang, JingJie Li, Cunqi Peng

**Affiliations:** a Department of Orthopedics, Hangzhou Lin’an Traditional Chinese Medicine Hospital, Hangzhou, Zhejiang Province, China; b Department of Orthopedics, Xiangyang Hospital of Traditional Chinese Medicine, Xiangyang, Hubei Province, China; c School of Acupuncture and Orthopedics, Hubei University of Chinese Medicine, Wuhan, Hubei Province, China.

**Keywords:** body roundness index, cross-sectional study, National Health and Nutrition Examination Survey, obesity, rheumatoid arthritis

## Abstract

This study aims to examine the association between body roundness index (BRI) and rheumatoid arthritis (RA) risk using data from National Health and Nutrition Examination Survey 1999 to 2020. A cross-sectional study of 33,239 adults was conducted. RA was identified through self-report, and BRI was calculated by standard formula. Weighted logistic regression models assessed the relationship between BRI and RA, adjusting for demographic and clinical factors. Restricted cubic spline and segmented regression models explored nonlinear trends. Higher BRI was significantly associated with increased RA risk (adjusted OR = 1.255 per unit increase, 95% CI: 1.204–1.307, *P* < .001). A nonlinear relationship was observed (*P* < .05). Subgroup analyses revealed stronger associations among nonsmokers and those with higher education. The area under the curve for BRI in predicting RA was 0.793 (95% CI: 0.785–0.802). Elevated BRI is independently associated with higher RA risk in a nonlinear, dose-response manner. BRI may serve as a practical marker for RA risk stratification and early intervention.

## 1. Introduction

Rheumatoid arthritis (RA) is characterized by synovitis, pannus formation, and subsequent destruction of articular cartilage and bone, ultimately resulting in joint deformities and functional impairment.^[1]^ According to the Global Burden of Disease analysis, approximately 17 million individuals worldwide are affected by RA, underscoring its significance as a global public health challenge. Furthermore, research suggests that the prevalence of RA is expected to rise due to population aging and increased life expectancy, with projections estimating that the global RA patient population could reach 31.7 million by 2050.^[2]^ This upward trend indicates that RA is not merely a medical condition but also places a substantial burden on social and economic systems. The onset of RA often precedes the clinical manifestation of synovitis. In 2012, the European League Against Rheumatism risk prediction study group delineated the progression of RA into 6 distinct stages^[3]^: 5 preclinical phases, including the genetic risk phase, environmental risk factor phase, systemic autoimmune dysregulation phase, asymptomatic arthritis phase, and undifferentiated arthritis phase, followed by the definitive diagnosis phase. Throughout this process, the body experiences an extended period of immune dysregulation. Timely interventions during this stage could potentially reverse immune imbalances, presenting a “therapeutic window of opportunity” to prevent the progression of RA.^[4]^ Nevertheless, current clinical practices have yet to achieve the early detection and treatment of RA. Therefore, identifying biomarkers associated with the pathogenesis of RA is essential to facilitate early diagnosis.

Obesity has been recognized as a significant risk factor for the development of RA. As a critical component of metabolic syndrome, obesity contributes to the progression of RA through various mechanisms.^[5–7]^ Specifically, it promotes systemic inflammation and immune dysfunction by activating pro-inflammatory cytokines, including tumor necrosis factor-α, interleukin-6, and interleukin-1β. Regular monitoring of obesity status may aid in the early identification of arthritis risk and facilitate better management of disease progression. Obesity is defined as a body mass index (BMI) ≥ 30 kg/m^2^, characterized by persistent metabolic disturbances, excessive fat accumulation, and metabolic abnormalities, making it a widely used criterion for diagnosing obesity-related conditions.^[8]^ However, BMI does not distinguish between subcutaneous fat and visceral fat, a limitation that has raised concerns about its clinical applicability.^[9]^ Visceral fat, which surrounds internal organs, is more strongly associated with metabolic diseases, insulin resistance, and elevated mortality risk than subcutaneous fat, even in individuals with a normal BMI.^[10]^ The body roundness index (BRI) has emerged as a novel metric for assessing obesity, offering significant advantages over traditional BMI and other conventional measures in evaluating fat distribution and related disease risks.^[11]^ Studies indicate that elevated BRI levels are strongly associated with metabolic syndrome,^[12]^ osteoarthritis,^[13]^ cardiovascular diseases,^[14]^ and depression,^[15]^ positioning it as an innovative tool for health assessment. While the specific relationship between BRI and RA remains unclear, existing evidence suggests that BRI may be linked to inflammatory diseases, providing a promising avenue for further investigation into its mechanisms. Utilizing data from the National Health and Nutrition Examination Survey (NHANES), this study conducted a cross-sectional analysis to systematically explore the association between BRI and RA for the first time. The findings aim to elucidate the potential link between BRI and RA, offering scientific insights and practical guidance for the prevention and management of RA.

## 2. Methods

### 2.1. Study design and population

**Figure 1.**
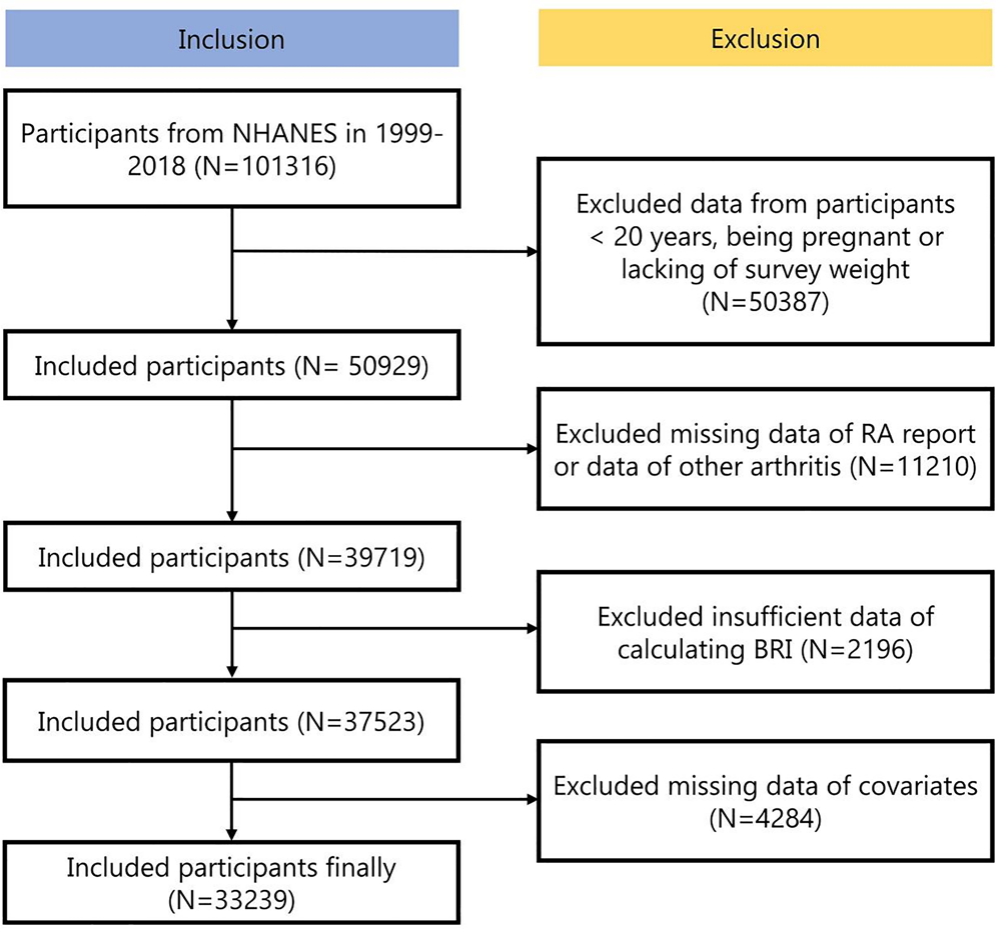
Screening flow of respondents.

The NHANES is a nationwide program designed to collect comprehensive data on the nutrition and health status of the U.S. population. Conducted biennially, NHANES employs a cross-sectional study design implemented through a complex, multistage probability sampling technique.^[16]^ This study utilized publicly available NHANES datasets, which were collected in accordance with ethical guidelines, with informed consent obtained from all participants. Detailed information on the study design and data collection methods is publicly accessible via the official platform (www.cdc.gov/nchs/nhanes/). All procedures adhered strictly to ethical standards and relevant regulations.^[17]^ This analysis utilized NHANES datasets from 1999 to 2020, initially including 101,316 participants. Exclusions were made as follows: 50,387 participants under 20 years of age, pregnant, or lacking weight data; 11,201 participants with incomplete data on RA; and 2196 participants with missing data, including key covariates (N = 4284). After applying these criteria, 33,239 eligible participants were included in the final analysis. Detailed results are presented in Figure 1 and Table S1 Supplemental Digital Content, https://links.lww.com/MD/Q947.

### 2.2. Study population

This study utilized NHANES data from 1999 to 2020 and employed a cross-sectional study design. Arthritis diagnosis was determined based on self-reported questionnaires, specifically survey items MCQ160a, MCQ191, and MCQ190. Participants were asked whether a doctor or other healthcare provider had ever informed them of an arthritis diagnosis, with response options of “Yes” or “No.” Additionally, participants were required to specify the type of arthritis they had, with possible responses including psoriatic arthritis, osteoarthritis, RA, other types, refusal to answer, or “don’t know.”

### 2.3. Body roundness index measurement

Anthropometric data, including body height, weight, and waist circumference (WC), were collected by professionally trained examiners at mobile examination centers equipped with standardized tools. Weight was measured using a calibrated platform scale with an accuracy of 0.1 kg, while height was recorded using a stadiometer in a standing position, accurate to 0.1 cm. These measurements were performed with participants wearing light clothing and no shoes to ensure accuracy. Consistent with prior studies, the BRI was calculated using the formula developed by Thomas et al^[18]^:


$$\begin{array}{l} 364.2-365.5*\sqrt{\frac{1-{\left[WC\left(m\right)/2\pi \right]}^{2}\;\;\;}{{\left[0.5*height\left(m\right)\right]}^{2}}}. \end{array}$$

### 2.4. Covariates

This study analyzed questionnaire and physical examination data from the NHANES database, including demographic information (age, gender, race/ethnicity, poverty income ratio [PIR], education level, and marital status) and behavioral factors (smoking and alcohol consumption). Race/ethnicity was self-reported and categorized as Mexican American, non-Hispanic White, non-Hispanic Black, and other races (including multiracial individuals). Education level was classified as less than high school, high school graduate, and more than high school, while marital status was grouped into married/cohabiting, widowed/divorced/separated, and never married. Smoking status was determined using a questionnaire, beginning with the question, “Have you smoked more than 100 cigarettes in your lifetime?” Participants responding “No” were classified as never smokers. Those answering “Yes” were further queried about current smoking behavior. Respondents indicating “Quit smoking” were classified as former smokers, while those stating “Still smoking” were classified as current smokers.^[19]^ Alcohol consumption was assessed using the question, “Do you drink alcohol at least 12 times per year?” A single drink was defined as 12 ounces of beer, 4 ounces of wine, or 1 ounce of spirits.^[20]^ Moderate alcohol consumption was defined as no more than 2 drinks per day for men and one drink per day for women. Exceeding these limits was categorized as excessive drinking, with men consuming more than 2 drinks and women consuming more than one drink per day. BMI data were derived from NHANES physical examination records^[21]^ and calculated using the formula calculated as BMI = *W*/*H*^2^ (weight in kilograms divided by height in meters squared), where weight (*W*) is measured in kilograms and height (*H*) in meters squared. BMI was classified according to U.S. population standards as underweight (≤25 kg/m^2^), overweight (25–30 kg/m^2^), and obese (≥30 kg/m^2^). Hypertension and diabetes were identified based on self-reported medical history from questionnaires.

### 2.5. Statistical analysis

Weighted statistical analyses were conducted for all participants, accounting for the complex multistage cluster sampling design of NHANES.^[22]^ Group differences for categorical variables were assessed using weighted χ^2^ tests, while differences for continuous variables were analyzed using weighted *t*-tests (ANOVA). To examine the association between BRI and RA, univariate and multivariate weighted logistic regression models were applied, incorporating three specific models: Model I (unadjusted for covariates), Model II (adjusted for age, gender, and race/ethnicity), and Model III (further adjusted for marital status, education level, PIR, smoking status, alcohol consumption, hypertension, cardiovascular disease, and diabetes history). Smoothing curve fitting was performed to evaluate potential nonlinear relationships between BRI and RA, with a two-piece linear regression model used to explore possible threshold effects.^[23]^ Subgroup analyses stratified by gender, age, and other key variables were conducted to investigate interactions between BRI and grouping variables on RA progression.^[24]^ The predictive performance of BRI for RA was assessed using receiver operating characteristic (ROC) curves and the area under the curve (AUC). All models were adjusted for relevant covariates, and statistical analyses were performed using R software (version 4.4.1), with sample interview weights applied to account for the survey design. Statistical significance was defined as a *P*-value < .05.

### 2.6. Statistical issues addressed

To address potential statistical concerns, we conducted comprehensive analyses.

#### 2.6.1. Multicollinearity assessment

We calculated Pearson correlation coefficients between BRI and BMI to assess potential collinearity.^[25]^ Given that BRI and BMI showed high correlation (*R* = 0.71, *P* < .001), we included only BRI in our final multivariable models to avoid multicollinearity issues. Variance inflation factors (VIF) were calculated for all variables in the final model, with VIF < 5 indicating acceptable collinearity levels^[26]^; Model diagnostics: Model fit was assessed using the Hosmer–Lemeshow goodness-of-fit test.^[27]^ We also performed residual analysis to check model assumptions.

#### 2.6.2. ROC analysis

Receiver operating characteristic curves were constructed using predicted probabilities from the fully adjusted model (Model 3) to evaluate the discriminative ability of BRI for RA prediction.^[28]^

#### 2.6.3. Subgroup and sensitivity analyses

Stratified analyses were performed by sex and age groups to assess the consistency of associations across different populations. Sensitivity analysis was conducted by excluding extreme BRI values (1st and 99th percentiles) to test the robustness of our findings.

## 3. Results

### 3.1. Characteristics of study objects

Table 1 summarizes the differences in characteristics between RA patients and non-RA participants. A total of 33,239 individuals were analyzed, among whom 6.8% (2274 participants) were diagnosed with RA. RA patients were generally older, with 43.9% aged ≥ 60 years, and a higher proportion were female (57.8%). RA patients also exhibited significantly higher rates of hypertension (53.4%), diabetes (18%), and obesity (BMI ≥ 30 kg/m^2^, 46.5%) compared to non-RA participants. They were more likely to have lower educational attainment and a higher prevalence of poverty (PIR ≤ 1.3, 31%). Additionally, RA patients had a higher rate of nonsmoking behavior (73.1%). Regarding racial distribution, RA patients included a greater proportion of non-Hispanic Black individuals (16.2%) and a lower proportion of Mexican Americans (6.2%). These findings indicate notable associations between RA and various demographic, socioeconomic, and health-related characteristics.

**Table 1:** Characteristics of Participants grouped by RA in NHANES 1999–2020.

Variables	Total	Non-RA group	RA group	*P*
n	33,239	30,965	2274	
Age, n (%)				
20–39	13,401 (44.2)	13,205 (46)	196 (12.3)	<.001
40–59	11,362 (38.5)	10,597 (38.3)	765 (43.8)	
≥60	8476 (17.2)	7163 (15.8)	1313 (43.9)	
Gender, n (%)				
Male	17,353 (51.5)	16,386 (52)	967 (42.2)	<.001
Female	15,886 (48.5)	14,579 (48)	1307 (57.8)	
Race, n (%)				
Mexican American	6033 (9)	5693 (9.1)	340 (6.2)	<.001
Other Hispanic	2740 (5.7)	2565 (5.7)	175 (4.6)	
Non-Hispanic White	14,054 (66.6)	13,082 (66.6)	972 (67.5)	
Non-Hispanic Black	7077 (11.4)	6410 (11.1)	667 (16.2)	
Other Race - including multi-racial	3335 (7.4)	3215 (7.5)	120 (5.6)	
Education, n (%)				
Less than high school	8239 (15.4)	7435 (14.9)	804 (24.1)	<.001
High school grad/GED or equivalent	7591 (23.4)	7026 (23.1)	565 (28.4)	
Higher than high school	17,409 (61.2)	16,504 (61.9)	905 (47.5)	
Marital status, n (%)				
Married/living with partner	20,298 (64)	19,059 (64.1)	1239 (61.1)	<.001
Widowed/divorced/separated	6331 (15.9)	5482 (15.1)	849 (31.2)	
Never married	6610 (20.1)	6424 (20.8)	186 (7.6)	
PIR, n (%)				
≤1.3	10,077 (21.2)	9171 (20.6)	906 (31)	<.001
1.3–3.5	12,554 (35.4)	11,704 (35.4)	850 (37)	
>3.5	10,608 (43.4)	10,090 (44)	518 (32)	
BMI, n (%)				
<25 kg/m^2^	10,776 (33.8)	10,249 (34.3)	527 (24.2)	<.001
25–30 kg/m^2^	11,325 (33.4)	10,633 (33.6)	692 (29.3)	
≥30 kg/m^2^	11,138 (32.8)	10,083 (32.1)	1055 (46.5)	
Smoking status, n (%)				
Current non smokers	25,947 (78.1)	24,237 (78.4)	1710 (73.1)	<.001
Current smokers	7292 (21.9)	6728 (21.6)	564 (26.9)	
Drinking status, n (%)				
Current non drinkers	11,372 (28.3)	10,321 (27.6)	1051 (40.5)	<.001
Current moderate drinkers	19,628 (63.7)	18,522 (64.3)	1106 (52.6)	
Current heavy drinkers	2239 (8.1)	2122 (8.1)	117 (6.8)	
Diabetes, n (%)				
Yes	3183 (6.8)	2656 (6.2)	527 (18)	<.001
No	30,056 (93.2)	28,309 (93.8)	1747 (82)	
Hypertension, n (%)				
Yes	9590 (25.1)	8255 (23.6)	1335 (53.4)	<.001
No	23,649 (74.9)	22,710 (76.4)	939 (46.6)	
BRI	5.07 (1.18)	5.04 (1.16)	5.71 (1.29)	

BMI = body mass index, BRI = body roundness index, GED = General Educational Development, NHANES = National Health and Nutrition Examination Survey, PIR = poverty income ratio, RA = rheumatoid arthritis.

### 3.2. Association between BRI and RA

Table 2 presents the associations between the BRI and the risk of RA across different models. In Model 1, which was unadjusted for covariates, each 1-unit increase in the continuous BRI variable was associated with a 2% increase in RA risk (OR = 1.02, 95% CI: 1.02–1.03, *P* < .001). Compared to the Q1 group, the odds ratios (ORs) for the Q2, Q3, and Q4 groups were 1.02 (95% CI: 1.02–1.03, *P* < .001), 1.04 (95% CI: 1.03–1.05, *P* < .001), and 1.07 (95% CI: 1.07–1.08, *P* < .001), demonstrating a significant dose-response relationship (*P* for trend < .001). In Model 2, which adjusted for age, gender, race, education level, PIR, and marital status, the OR for the continuous BRI variable increased to 1.32 (95% CI: 1.25–1.40, *P* < .001). The ORs for Q2, Q3, and Q4 groups were 1.49 (95% CI: 1.17–1.89, *P* < .001), 1.87 (95% CI: 1.48–2.36, *P* < .001), and 2.50 (95% CI: 1.99–3.13, *P* < .001), further indicating that higher BRI significantly increased RA risk. In Model 3, fully adjusted for all covariates – including BMI, smoking status, alcohol use, diabetes, and hypertension – the OR for the continuous BRI variable was 1.22 (95% CI: 1.11–1.33, *P* < .001). The ORs for Q2, Q3, and Q4 groups were 1.44 (95% CI: 1.12–1.86, *P* = .005), 1.65 (95% CI: 1.23–2.20, *P* < .001), and 1.76 (95% CI: 1.28–2.41, *P* < .001), respectively. In all models, the *P* for trend remained significant (*P* < .001), indicating a robust positive correlation between BRI and RA risk.

**Table 2:** Associations between BRI and RA in weighted logistic regression models.

	Model 1		Model 2		Model 3	
	OR (95% CI)	*P*	OR (95% CI)	*P*	OR (95% CI)	*P*
BRI						
Continuous	1.02 (1.02–1.03)	<.001	1.32 (1.25–1.40)	<.001	1.22 (1.11–1.33)	<.001
Q1	Reference				Reference	
Q2	1.02 (1.02–1.03)	<.001	1.49 (1.17–1.89)	<.001	1.44 (1.12–1.86)	.005
Q3	1.04 (1.03–1.05)	<.001	1.87 (1.48–2.36)	<.001	1.65 (1.23–2.20)	<.001
Q4	1.07 (1.07–1.08)	<.001	2.50 (1.99–3.13)	<.001	1.76 (1.28–2.41)	<.001
*P* for trend	<.001		<.001		<.001	

Model 1 was not adjusted for any covariate; Model 2 was adjusted for age, gender, race, education, PIR, marital status; Model 3 was adjusted for age, gender, race, education, PIR, marital status, BMI, smoking status, drinking status, diabetes and hypertension.

BRI and BMI showed high correlation (*R* = 0.71), therefore only BRI was included in multivariable models to avoid multicollinearity. All VIF values in Model 3 were < 5.

BMI = body mass index, BRI = body roundness index, CI = confidence interval, OR = odds ratio, PIR = poverty income ratio, RA = rheumatoid arthritis, VIF = variance inflation factors.

Before conducting multivariable analyses, we assessed the correlation between BRI and BMI. The Pearson correlation coefficient was 0.71 (*P* < .001), indicating high correlation. To avoid multicollinearity, we included only BRI in our multivariable models. In the final adjusted model, all VIF values were <5, confirming no severe multicollinearity issues.

The Hosmer–Lemeshow test indicated some model fit concerns (*P* ≈ 0), suggesting potential for model optimization. However, the consistent associations across different model specifications support the robustness of our findings.

### 3.3. The associations between BRI and RA syndrome stages in RCS regression model

**Figure 2.**
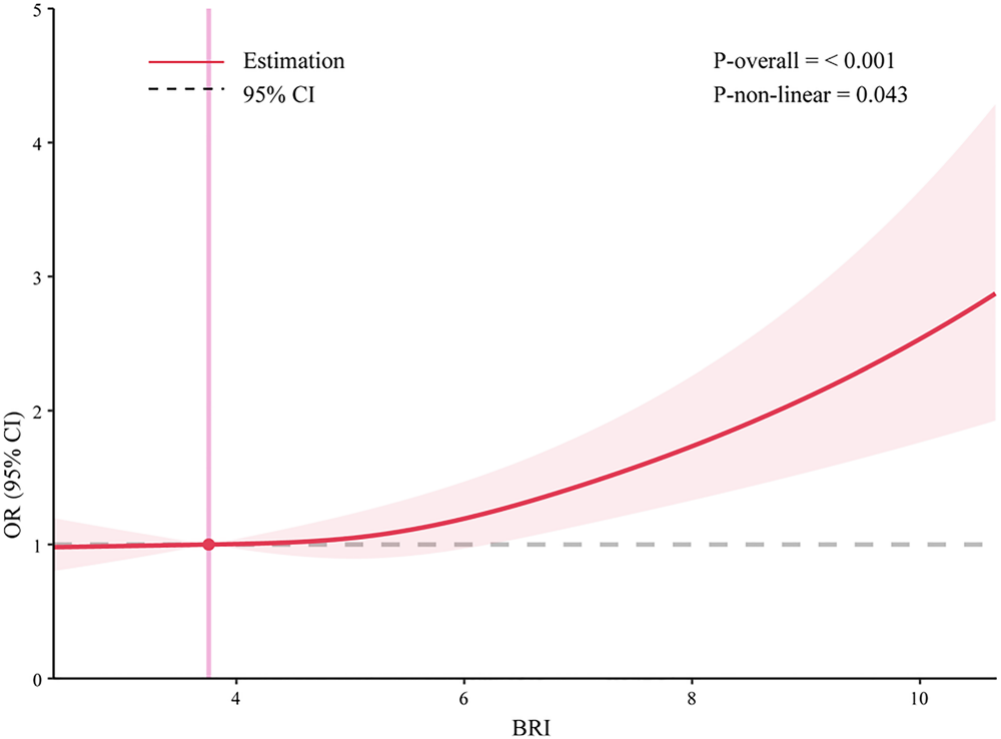
RCS curve of the association between BRI and RA among all participants. It was adjusted for age, gender, race, education, PIR, marital status, BMI, smoking status, drinking status, diabetes and hypertension. BMI = body mass index, BRI = body round index, OR = odds ratio, PIR = poverty income ratio, RA = rheumatoid arthritis, RCS = restricted cubic spline.

The dose-response relationship shows a significant association between BRI and the risk of RA (Fig. 2). Specifically, there is a nonlinear relationship between BRI and RA, as indicated by the overall *P* value (*P*_overall < .05) and the nonlinear *P* value (*P*_non-linear = .043). Moreover, as BRI increases, the risk of developing RA also gradually increases.

### 3.4. Subgroup analysis

**Figure 3.**
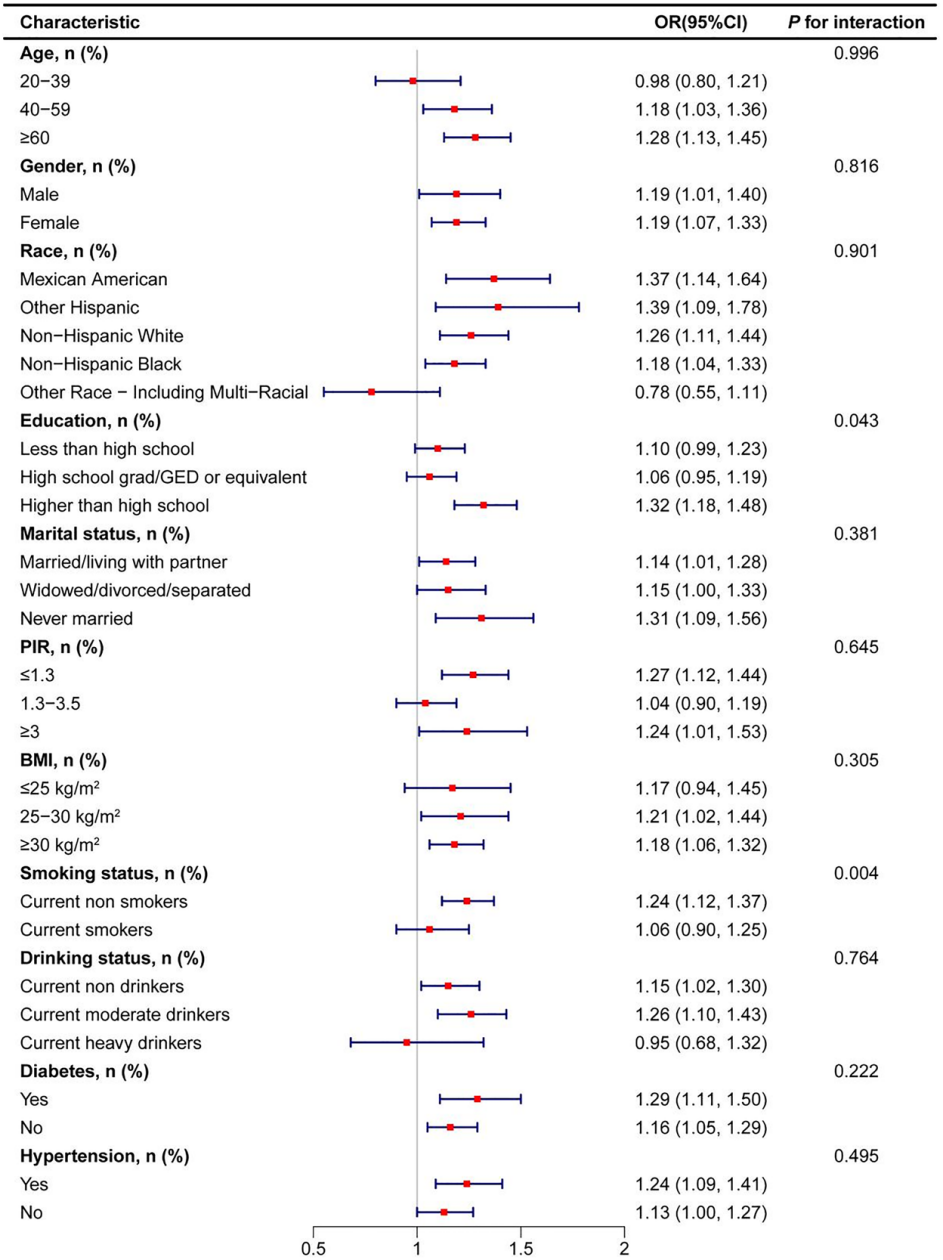
Subgroup analyses of the associations between BRI and RA. BRI = body round index, RA = rheumatoid arthritis.

To evaluate the stability of the positive correlation between BRI and the risk of RA, participants were stratified into different subgroups based on sociodemographic characteristics and health status, and the associations were analyzed within each subgroup (see Fig. 3 and Table S2, Supplemental Digital Content, https://links.lww.com/MD/Q947). The subgroup analysis results indicated that the association between BRI and RA risk was significant across most characteristics. Notably, in the smoking status subgroup, a significant interaction between BRI and RA risk was observed (*P* for interaction = .004), suggesting that smoking may modulate this relationship. Specifically, in nonsmokers, the association between BRI and RA risk was stronger (OR = 1.24, 95% CI: 1.12–1.37), while in smokers, the association was weaker (OR = 1.06, 95% CI: 0.90–1.25). Additionally, a significant interaction was detected in the education level subgroup (*P* for interaction = .043). Among participants with higher education levels, the association between BRI and RA risk was the most pronounced (OR = 1.32, 95% CI: 1.18–1.48). Although the interaction *P* values in other subgroups (such as age, gender, race, marital status, PIR, BMI, alcohol consumption, diabetes, and hypertension) did not reach statistical significance, they still demonstrated a consistent positive correlation trend. These findings suggest that the association between BRI and RA risk remains robust across different sociodemographic characteristics and health statuses, with smoking status and education level potentially serving as important factors influencing this relationship.

### 3.5. Predictive value of BRI on RA

**Figure 4.**
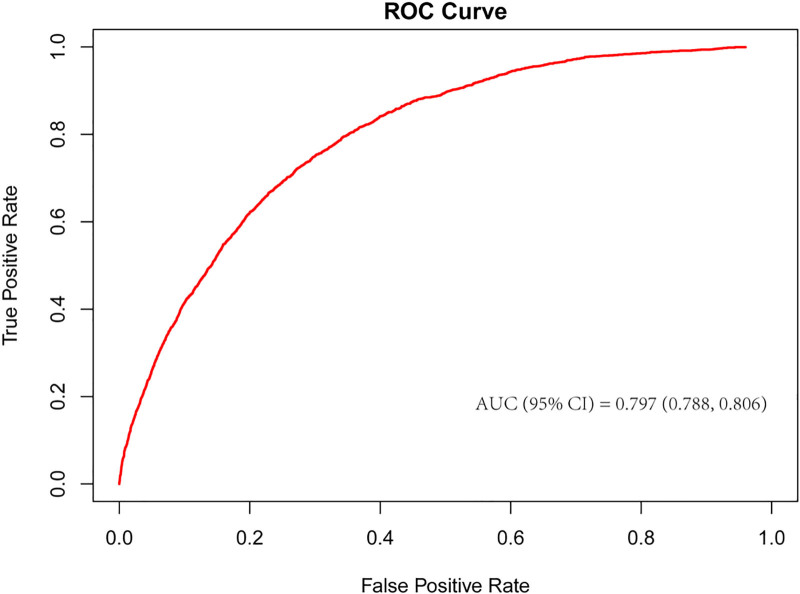
ROC curve of the predictive value for BRI on RA using the fully adjusted model (Model 3). AUC = area under the curve, BRI = body round index, RA = rheumatoid arthritis, ROC = receiver operating characteristic.

We validated the predictive performance of the BRI for RA risk using ROC curve analysis. ROC curve analysis using the fully adjusted model (Model 3) showed an AUC of 0.793 (95% CI: 0.785–0.802) for predicting RA risk (Fig. 4). Figure 4 shows the ROC curve constructed using predicted probabilities from the fully adjusted multivariable logistic regression model (Model 3). The optimal cutoff value was 0.0652, corresponding to a sensitivity of 75.8% and specificity of 69.3%. Model comparison using DeLong test showed significant improvement from the crude model (Model 1) to the fully adjusted model (*P* < .001). The study suggests that BRI has good predictive ability for RA risk.

### 3.6. Sensitivity analysis

To test the robustness of our findings, we conducted sensitivity analysis by excluding participants with extreme BRI values (1st and 99th percentiles). After excluding 666 participants, the association remained significant in the remaining 32,573 participants (adjusted OR = 1.257, 95% CI: 1.201–1.316, *P* < .001), confirming the stability of our results. The results are shown in Table 3.

**Table 3:** Subgroup analysis of associations between BRI and RA.

Subgroup	Sample_Size	RA_Cases	OR (95% CI)	*P*_value
Sex				
Male	17,353	967	1.302 (1.212–1.397)	<.001
Female	15,886	1307	1.221 (1.161–1.284)	<.001
Age group				
Age ≤ 2	24,763	961	1.271 (1.203–1.342)	<.001
Age = 3	8476	1313	1.253 (1.181–1.331)	<.001

Age groups represent categorical coding in NHANES dataset where 1 = youngest, 2 = middle, 3 = oldest age categories. Adjusted for race, education, PIR, marital status, smoking, drinking, diabetes, and hypertension.

BRI = body roundness index, CI = confidence interval, NHANES = National Health and Nutrition Examination Survey, OR = odds ratio, PIR = poverty income ratio, RA = rheumatoid arthritis.

## 4. Discussion

In this cross-sectional study using a nationally representative sample, we examined the relationship between BRI and RA. Our results demonstrate a significant association between BRI and RA (adjusted OR = 1.255, 95% CI: 1.204–1.307), even after adjusting for potential confounding factors. The model showed good discriminative ability with an AUC of 0.793. BRI may function as a predictive biomarker for RA, offering important insights for prevention and treatment strategies. Managing weight, reducing WC, and consequently lowering BRI levels could potentially reduce the risk of developing RA.

This study is the first to directly investigate the relationship between BRI and RA. Previous research has shown that obesity can induce systemic chronic low-grade inflammation, immune system dysfunction, and excessive production of reactive oxygen species, all of which may contribute to the onset and progression of RA.^[29,30]^ Our findings reveal a significant positive correlation between elevated BRI levels and increased RA prevalence. As an effective indicator of abdominal obesity and its associated health risks, these results align with expectations, suggesting that abdominal obesity may represent a potential risk factor for RA. However, the precise relationship between increased BRI and RA pathogenesis remains incompletely understood. BRI, which assesses the degree of abdominal fat accumulation and metabolic disturbances, is closely associated with RA development. Its pathogenic mechanisms involve multiple factors. First, visceral fat in obesity acts as an active endocrine organ, secreting various pro-inflammatory cytokines such as tumor necrosis factor-α, interleukin-6, and IL-1. These cytokines activate critical inflammatory signaling pathways, including NF-κB and JNK, driving chronic low-grade systemic inflammation. Additionally, chemokines attract macrophage infiltration into the synovium, worsening local inflammation.^[31]^ Second, metabolic disturbances in fat metabolism result in elevated levels of free fatty acids and lipopolysaccharides (LPS), which bind to toll-like receptors and activate the NLRP3 inflammasome. This leads to increased interleukin-1β release, enhancing inflammation and exacerbating immune dysfunction.^[32,33]^ Moreover, visceral fat in obesity promotes the differentiation of Th17 cells, which secrete IL-17, a critical factor driving synovial inflammation and bone destruction in RA.^[34,35]^ Simultaneously, obesity significantly impairs the immune-suppressive function of regulatory T cells (Tregs), reducing the body’s ability to control abnormal immune responses and further increasing the risk of RA development.^[36,37]^ The exacerbation of local synovial inflammation also plays a key role in linking obesity with RA. In obese individuals, adipose tissue may directly infiltrate the synovium, releasing local pro-inflammatory cytokines, thus intensifying immune responses and tissue damage in the synovium.^[38]^ Furthermore, insulin resistance, a hallmark of obesity, creates a vicious cycle with pro-inflammatory responses, further activating the inflammatory cascade and aggravating the pathological progression of RA.^[39]^

It is crucial to recognize that smoking and education level may modulate the relationship between BRI and RA through distinct pathways. Smoking, in particular, may exacerbate the RA risk associated with BRI via direct physiological mechanisms. Smoking induces systemic inflammation and oxidative stress, activates the immune system, and amplifies the chronic inflammatory burden associated with obesity.^[40,41]^ Additionally, smoking may alter fat distribution and metabolic characteristics, promoting central obesity and further increasing the risk of RA through dysregulated adipokine secretion.^[42,43]^ In contrast, education level primarily influences this relationship through behavioral and socioeconomic pathways. Individuals with lower education levels often engage in unhealthier lifestyles, such as smoking, poor dietary habits, and lack of physical activity, which contribute to increased obesity and inflammation.^[44]^ For those with high BRI, weight management may be more challenging within the low-education group, heightening the likelihood of RA development. Furthermore, individuals with lower education levels often face limited access to health education and medical resources, potentially delaying early interventions for RA. The lack of awareness regarding obesity and associated risk factors among these populations may intensify the impact of BRI on RA risk.^[45,46]^

The BRI is an innovative anthropometric measure that has demonstrated notable advantages in identifying obesity – particularly abdominal obesity – and predicting associated health risks. Unlike traditional BMI, which relies solely on height and weight, BRI incorporates height, WC, and weight, enabling a more nuanced evaluation of body shape and fat distribution.^[47]^ This distinction is particularly valuable as BRI emphasizes WC, offering superior sensitivity and accuracy in detecting visceral and abdominal fat accumulation. Consequently, it provides a more precise assessment of risks associated with abdominal obesity and metabolic diseases. Additionally, BRI’s reduced reliance on muscle mass makes it a reliable health indicator even for individuals with high muscle mass.^[48,49]^ One of BRI’s most significant advantages is its applicability across diverse populations, including variations in gender, age, and racial groups.^[50]^ Studies indicate that BRI can capture differences in fat distribution and related health risks more effectively across these demographic categories, offering a tailored risk assessment for diverse populations. Furthermore, the integrated approach of BRI, combining multiple body measurements, minimizes errors inherent to single-measurement methods and enhances its predictive power for metabolic disorders such as diabetes,^[51]^ bone density issues,^[52]^ and hypertension.^[53]^ Importantly, BRI serves as an early warning system for health risks related to insulin resistance and chronic inflammation.^[54,55]^ In practical applications, BRI excels in monitoring weight management and evaluating the effectiveness of health interventions.^[56,57]^ Its straightforward calculation, requiring only basic measurements like height and WC, avoids the need for complex equipment, thereby reducing operational costs.^[58]^ This feature makes BRI especially valuable for monitoring obesity-related diseases in resource-limited settings. Additionally, its technical precision and affordability position it as a vital tool for health management and disease prevention.^[59]^ In conclusion, BRI is a multifunctional and practical anthropometric indicator with significant potential to enhance the management and prevention of obesity-related diseases. Its technical accuracy, predictive capabilities, and cost-effectiveness offer robust support for advancing public health and personalized healthcare. With continued research, BRI’s role in addressing obesity and its associated health risks is likely to expand, contributing to improved outcomes in global health management.

This study has several notable strengths, particularly the use of the NHANES database, which provides high-quality, nationally representative, and diverse data. NHANES collects data using standardized methods and includes a comprehensive array of health indicators, ranging from chronic conditions to dietary behaviors. This robust dataset forms a solid foundation for health research and public health policy. Additionally, the database’s long history and open accessibility make it invaluable for analyzing health trends and supporting multidisciplinary research efforts. Moreover, this study conducted subgroup stratification analyses, revealing the significant relationship between the BRI and RA across diverse populations. This approach enhances the reliability and generalizability of the findings, shedding light on how the association varies across different demographic and health-related characteristics. However, the study is not without limitations. First, the cross-sectional design of NHANES restricts the ability to establish causal relationships; thus, the findings primarily reflect associations rather than causality. Second, NHANES lacks detailed regional information, which limits its utility for addressing localized policy needs or interventions. Additionally, certain specific subgroups had limited sample sizes, which may constrain the ability to perform in-depth analyses of unique populations or uncover nuanced relationships. To address these limitations, future research should integrate NHANES data with other datasets, such as longitudinal studies or region-specific health surveys, to enable more comprehensive analyses and stronger causal inferences. Expanding sample sizes in underrepresented subgroups and incorporating more granular geographic data could also strengthen the relevance and applicability of the findings.

Additionally, while we addressed multicollinearity between BRI and BMI by including only BRI in our models, future studies should explore the independent contributions of different anthropometric measures. The Hosmer–Lemeshow test results suggest potential for further model optimization, though the consistent associations across different model specifications support our findings. Furthermore, the age variable in our dataset was categorically coded rather than continuous, which may limit precise assessment of age effects.

In conclusion, this study investigated the relationship between the BRI and RA, uncovering a significant association between higher BRI and an elevated risk of RA. These findings suggest that BRI could serve as a novel predictive indicator for RA, providing a more accurate assessment of abdominal obesity and associated health risks compared to traditional measures like BMI. This research offers a valuable foundation for developing early detection and preventive strategies targeted at high-risk populations. Clinicians may consider incorporating BRI into comprehensive screening protocols to better identify individuals at an increased risk of RA, enabling earlier intervention and tailored management approaches. However, further large-scale, high-quality studies are essential to validate these findings and deepen our understanding of the role BRI plays in RA pathogenesis. Such research could refine the application of BRI in clinical practice and enhance strategies for preventing and managing RA.

Our analysis addressed several important statistical considerations. The high correlation between BRI and BMI (*R* = 0.71) necessitated including only BRI in our multivariable models to avoid multicollinearity, which is supported by VIF values <5. The cross-sectional design limits our ability to infer causality, and our findings should be interpreted as associations rather than causal relationships. The subgroup analyses demonstrated consistent associations across different demographic groups, supporting the robustness of our findings. Additionally, sensitivity analysis excluding extreme values confirmed the stability of the BRI-RA association.

## Author contributions

**Conceptualization:** Wenzhuo Huang.

**Data curation:** JingJie Li.

**Visualization:** Cunqi Peng.

**Writing – original draft:** Xin Huang.

## Supplementary Material


